# Arsenic Exposure in Pregnancy Increases the Risk of Lower Respiratory Tract Infection and Diarrhea during Infancy in Bangladesh

**DOI:** 10.1289/ehp.1002265

**Published:** 2010-12-09

**Authors:** Anisur Rahman, Marie Vahter, Eva-Charlotte Ekström, Lars-Åke Persson

**Affiliations:** 1 International Centre for Diarrhoeal Disease Research, Bangladesh, Dhaka, Bangladesh; 2 International Maternal and Child Health, Department of Women’s and Children’s Health, Uppsala University, Uppsala, Sweden; 3 Institute of Environmental Medicine, Karolinska Institutet, Stockholm, Sweden

**Keywords:** arsenic, Bangladesh, diarrhea, infants, pregnancy, respiratory tract infection

## Abstract

**Background:**

Previous studies have reported associations between prenatal arsenic exposure and increased risk of infant mortality. An increase in infectious diseases has been proposed as the underlying cause of these associations, but there is no epidemiologic research to support the hypothesis.

**Objective:**

We evaluated the association between arsenic exposure in pregnancy and morbidity during infancy.

**Methods:**

This prospective population-based cohort study included 1,552 live-born infants of women enrolled during 2002–2004 in Matlab, Bangladesh. Arsenic exposure was assessed by the concentrations of metabolites of inorganic arsenic in maternal urine samples collected at gestational weeks 8 and 30. Information on symptoms of lower respiratory tract infection (LRTI) and diarrhea in infants was collected by 7-day recalls at monthly home visits.

**Results:**

In total, 115,850 person-days of observation were contributed by the infants during a 12-month follow-up period. The estimated risk of LRTI and severe LRTI increased by 69% [adjusted relative risk (RR) = 1.69; 95% confidence interval (CI), 1.36–2.09)] and 54% (RR = 1.54; 95% CI, 1.21–1.97), respectively, for infants of mothers with urinary arsenic concentrations in the highest quintile (average of arsenic concentrations measured in early and late gestation, 262–977 μg/L) relative to those with exposure in the lowest quintile (< 39 μg/L). The corresponding figure for diarrhea was 20% (RR = 1.20; 95% CI, 1.01–1.43).

**Conclusions:**

Arsenic exposure during pregnancy was associated with increased morbidity in infectious diseases during infancy. Taken together with the previous evidence of adverse effects on health, the findings strongly emphasize the need to reduce arsenic exposure via drinking water.

Arsenic is a highly potent toxicant and carcinogen [International Agency for Research on Cancer 2004; National Research Council (NRC) 2001]. Worldwide, millions of individuals are drinking well water with arsenic levels above the World Health Organization (WHO) guideline value of 10 μg/L (Kinniburg 2001; Nordstrom 2002; WHO 2004). Arsenic concentrations are high in certain geologic formations, and arsenic can dissolve easily to groundwater and contaminate local tube wells and other public water supplies. This has been a problem in many parts of the world, including Argentina, Bangladesh, Chile, China, Hungary, India, Taiwan, and parts of the United States (Chowdhury et al. 2000; Nordstrom 2002; Smith 2000). Arsenic exposure is associated with several severe health consequences, such as cancers, chronic diseases, and possibly with negative reproductive outcomes (NRC 2001).

An ecologic study in Chile reported increased neonatal and postneonatal mortality in an area with high arsenic content in the public water supply (Hopenhayn-Rich et al. 2000). In a retrospective cohort of about 29,000 women in Bangladesh, we found a significant dose-dependent association between infant mortality and arsenic exposure measured in tube-well water used by pregnant women (Rahman et al. 2007). The association was more pronounced when the outcome was restricted to infectious disease deaths. We also observed a significant increase risk of infant mortality in association with urinary arsenic concentrations during pregnancy in a prospective cohort study conducted in the same area (Rahman et al. 2010).

Arsenic-related infant mortality may, in part, be mediated via infectious diseases. A few animal and human studies have indicated that arsenic is associated with immunosuppression. In experimental studies, arsenic exposure suppressed the immunoglobulin (Ig) M and IgG antibody–forming cell response (Selgrade 2007), decreased interleukin-2 mRNA expression (Conde et al. 2007), and inhibited antigen-driven T-cell proliferation and macrophage activity and suppressed contact hypersensitivity responses (Patterson et al. 2004). Further, inorganic arsenic was found to impair the function of human macrophages both *in vivo* and *in vitro* (Banerjee et al. 2009; Lemarie et al. 2006). In a study in Mexico, an increased concentration of arsenic in urine was associated with reduced proliferative response to phytohemagglutinin stimulation, CD4^+^ subpopulation proportion, and interleukin-2 secretion levels in children (Soto-Pena et al. 2006). We recently reported an association between arsenic exposures through drinking water during pregnancy in rural Bangladesh and reduced placental T cells and altered cord blood cytokines, indicating effects of arsenic on immune function (Ahmed et al. 2011). These immunologic effects may potentially contribute to a high burden of infectious diseases in childhood.

Lower respiratory tract infection (LRTI) and diarrhea are two of the most common causes of morbidity and mortality in children < 5 years of age, especially in low-income countries. So far, very little is known about the influence of arsenic exposure on these morbidities. The objective of this prospective cohort study was to evaluate the association between prenatal arsenic exposure and LRTI and diarrhea during infancy in Bangladesh.

## Methods

### Study site

The study was conducted at Matlab, a subdistrict of Chandpur, Bangladesh, where arsenic contamination in tube-well water is common (Rahman et al. 2006). At the study site, the International Centre for Diarrhoeal Disease Research, Bangladesh (ICDDR,B), has been running a health and demographic surveillance system (HDSS) since 1966 in a population of 220,000. The HDSS area is divided into two parts: the ICDDR,B service area, where ICDDR,B provides services to all women of reproductive age and their children < 5 years of age; and the government service area, where health services are provided at government facilities. The ICDDR,B service area comprises four administrative blocks that each has a subcenter where paramedical staff provide 24-hr maternal and child health care. All subcenters are linked to a hospital, located at Matlab Township. Community health research workers (CHRWs), employed by the HDSS, collect information on vital events such as marriage, birth, death, and migration during monthly household visits with area residents.

### Study design and participants

This prospective cohort study was embedded into a food and micronutrient supplementation trial: Maternal and Infant Nutrition Interventions, Matlab (MINIMat trial, ISRCTN 16581394). The study was based on a cohort of live-born infants of pregnant women who were enrolled in the MINIMat study from February 2002 to January 2003, and data collection in the field continued until the end of 2004. Enrollment in the MINIMat study has been described elsewhere (Rahman et al. 2009). In short, during monthly home visits CHRWs performed urine pregnancy tests for women who reported that their menstrual period was > 2 weeks late. Women with a positive pregnancy test were invited to donate the remaining urine sample, which was usually collected at or around gestational week (GW) 8 for arsenic analysis. All women with a positive pregnancy test were advised to visit their health clinic for further evaluation of eligibility for the MINIMat study: a viable fetus < GW13 as detected by ultrasound examination, no severe illness, and consent for participation. On enrollment, usually around GW9, pregnant women were randomly assigned to receive an early invitation to begin food supplementation (started in the first trimester) or an invitation at the usual time (started in the second trimester). Women in each group were further randomized into three micronutrient supplementation groups: *a*) 30 mg iron and 400 μg folic acid, *b*) 60 mg iron and 400 μg folic acid, or *c*) the United Nations Children’s Fund/United Nations University/WHO preparation of 15 different micronutrients, including 30 mg iron and 400 μg folic acid. Food supplementation continued until the pregnancy was completed; micronutrient supplementation was continued until 6 weeks postpartum. For the present study, an additional urine sample was collected from all enrolled women during a routine clinic visit late in gestation, usually at or around GW30.

Mother–infant pairs were included in the present analysis if they had at least one postnatal monthly visit (to collect morbidity information) and had measured maternal urinary arsenic concentrations at early and late gestation. Of 2,281 women enrolled in the MINIMat study between February 2002 and January 2003, 1,552 were available for analysis. In total, 729 women were not available for evaluation because of abortion (*n* = 164), stillbirth (*n* = 42), loss to follow-up (including outmigration; *n* = 361), and no urine arsenic concentration measurement at GW8 or GW30 (*n* = 162). Because each mother–infant pair was followed up every month in infancy, and each visit included a 7-day morbidity recall, an infant could contribute up to 84 days to the study base. The mean number of person-days of observation was 75. In total, 115,850 person-days of observation were contributed by 1,552 infants.

### Exposure assessment

Individual exposure was assessed based on urinary concentration of the sum of inorganic arsenic and the methylated metabolites, methylarsonic acid, and dimethylarsinic acid (DMA), in early and late gestational periods. Individual exposure was also assessed by average of the two urinary arsenic concentrations (in early and late gestation). Details concerning sample collection and transport have been described elsewhere (Vahter et al. 2006). In brief, CHRWs collected GW8 urine samples during monthly home visits. The samples were chilled immediately with cooling blocks and transported to Matlab Hospital laboratory, where the samples were stored at −70°C. GW30 urine samples were collected during antenatal visits to the subcenters. These samples were initially stored at −20°C until end of the day, when they were transported to Matlab Hospital and stored at −70°C. Batches of samples were then transported frozen to Karolinska Institutet (Stockholm, Sweden), where they were analyzed using hydride generation atomic absorption spectroscopy (Vahter et al. 2006). The detection limit was 1.3 ± 0.27 μg/L. A couple of urine samples that contained arsenic concentrations close to the detection limit were reanalyzed using a larger volume; thus, no samples had arsenic concentrations below the detection limit. Longitudinal quality control showed good analytical quality (Vahter et al. 2006). Urinary arsenic concentrations were adjusted by specific gravity (average, 1.012 g/mL) to account for differences in urine dilution due to variation in fluid intake, time of sampling, temperature, and physical activity (Nermell et al. 2008). The concentration of metabolites of inorganic arsenic in urine is a recognized biomarker of the exposure to inorganic arsenic (Vahter 2002). Additional exposure to DMA or to organic arsenic metabolized in the body to DMA, which usually is attributable to consumption of seafood such as bivalves and seaweeds, was considered minimal because such seafoods are essentially never eaten in this area.

### Outcomes

A birth notification system was developed for the MINIMat study, and mother–infant pairs were followed up by monthly home visits. Information on symptoms related to LRTI and diarrhea, the two most common morbidities during infancy, was collected by repeated 7-day recalls. Mothers were asked about cough and/or difficult breathing in their infants in the preceding 7 days. If a woman’s answer was positive, she was further asked about the presence of rapid respiration and chest in-drawing (recession of subcostal margin during inspiration). The mother was also asked about passage of loose or watery stools, including frequency or passage of stool with blood. LRTI was defined as cough and/or difficult breathing combined with rapid respiration. Severe LRTI was defined as symptoms of LRTI accompanied by chest in-drawing. Diarrhea was defined as three or more liquid stools in 24 hr or stools mixed with blood.

### Covariates

At enrollment in the MINIMat study, information on women’s age, parity, education, and socioeconomic status was collected. Education was expressed as number of completed years in formal school. Parity was defined as the number of previous live births. Economic status was assessed by generating scores through principal component analysis based on household assets, housing structure, land occupation, and income. The generated scores were thereafter indexed into quintiles, where 1 represents the poorest and 5 the richest (Gwatkin et al. 2000). Last menstrual period was determined by recall at the time the pregnancy test conducted. Gestational age was calculated by subtracting last menstrual period date from the date of birth of infants and was expressed in weeks.

Women’s weight and height were measured at the first clinic visit during pregnancy around GW9. Weight was measured by electronic scales (model UNISCALE; SECA, Hamburg, Germany; precision of 100 g), and height was measured with a locally made wooden scale (precision of 0.1 cm). Body mass index (BMI), used as a measure of the nutritional status of the women, was calculated as weight divided by height squared (kilograms per square meter).

The birth notification system enabled measurement of birth anthropometry within 72 hr. Data on sex of the child and birth weight were collected. Birth weight was measured by beam scales (model 770; SECA) with a precision of 10 g.

### Statistical analysis

Incidence rates with 95% confidence intervals (CIs) were estimated for LRTI and diarrhea morbidity in the infant population as a whole. Median urinary arsenic concentrations (micrograms per liter) were estimated according to the background characteristics, and *p*-values for associations were estimated by median test. Associations between outcomes and background characteristics were also assessed, and *p*-values for associations were estimated by the chi-square test. Statistical associations were determined based on *p* < 0.05 significance level. Relative risks (RRs) of LRTI, severe LRTI, and diarrhea in relation to average prenatal arsenic exposure (mean arsenic concentrations of early and late gestation) were evaluated by Poisson regression, with arsenic exposure divided into quintiles and the first quintile used as the reference group. We also estimated associations with early and late exposures separately based on measurements of samples taken during early and late pregnancy. We assessed the dose–response relationship of arsenic exposure (by quintile of arsenic exposure as an ordinal variable) with risk of morbidity.

In addition, we performed stratified analyses on important social and biological strata to evaluate the robustness of the findings in the different models. We tested for presence of effect modification by making an interaction term (exposure × independent variable) and entering the term along with exposure and variable of interest in the model.

We identified potential confounders on the basis of associations with both the exposure and outcome at *p* < 0.20 and were included in the multivariate model if they changed the RR for arsenic by ≥ 5% or were associated with outcomes with *p* < 0.10.

### Ethical consideration

The MINIMat study obtained informed written consent from mothers for participation in the study for themselves and also for their infants. Because of the long time period between the collection of urine samples and laboratory analysis, we were not able to inform participants about their individual arsenic exposure. However, a separate project was initiated in parallel that assessed arsenic concentrations in water from all tube wells in the area and used red paint to identify unsafe tube wells with arsenic concentrations above the local drinking water standard of 50 μg/L (Rahman et al. 2006). In addition, different options for safe water sources were discussed during a series of community meetings conducted as part of the mitigation activities. This study was approved by the ethical review committees of ICDDR,B and Karolinska Institutet.

## Results

The mean (± SD) age of the women included in the analysis was 27 ± 6 years at enrollment. About one-third of the women were nulliparous, and one-fourth had not completed even 1 year at school. The mean body weight and height of women at GW8 were 45.0 ± 6.6 kg and 149.6 ± 5.2 cm, respectively, and about one-third of women were malnourished (BMI < 18.5 kg/m^2^). The mean duration of pregnancy was 39 ± 2 weeks. About 12% of the infants were born preterm (< 37 weeks). The mean birth weight of infants was 2,688 ± 398 g, and 52% were male. In this cohort, women did not smoke cigarettes or consume alcohol.

Table 1 presents the concentrations of arsenic in urine at GW8 and GW30 and the average of those two measures. We found wide variations of arsenic concentrations in maternal urine. The urinary arsenic concentrations at GW8 and GW30 were significantly correlated (Spearman’s *r* = 0.61; *p* ≤ 0.001).

The incidence rates of LRTI and severe LRTI in the infants during their first year of life were 2.96 (95% CI, 2.78–3.16) and 2.35 (95% CI, 2.18–2.52) episodes/person-year, respectively. The incidence of diarrhea was 4.01 (95% CI, 3.80–4.24) episodes/person-year.

Mothers’ education, socioeconomic status by asset index, and BMI were associated with urinary arsenic concentrations. Maternal parity, education, asset index, and gestational age at birth were associated with both LRTI and diarrhea morbidities (Table 2). BMI and sex of infant were associated with LRTI only, whereas maternal age was associated with diarrhea only (Table 2). The randomized food and micronutrient treatment groups of the MINIMat trial were not associated with arsenic exposure levels (*p* = 0.638 by Kruskal–Wallis test).

Arsenic exposure was significantly associated with the risk of LRTI and diarrhea (Table 3). Compared with exposure to average urinary arsenic at the first quintile level, the risk of LRTI and severe LRTI was increased by 69% (RR 1.69; 95% CI, 1.36–2.09) and 54% (RR = 1.54; 95% CI, 1.21–1.97), respectively, for women who had urinary arsenic concentrations at the fifth quintile level. The corresponding increase for diarrhea was 20% (RR = 1.20; 95% CI, 1.01–1.43). We also observed dose-dependent associations of arsenic exposures with both LRTI and severe LRTI across quintiles of arsenic concentrations at GW8, GW30, and the average arsenic concentrations (test for linear-trend *p* < 0.05). We observed a dose-dependent association of risk of diarrhea with arsenic concentration at GW8 and the average concentrations, but not with arsenic concentrations at GW30 (test for linear-trend *p* > 0.05).

Comparison of the impact of arsenic exposure in early and late gestation on morbidity showed that the risk for LRTI was more pronounced if the exposure was based on urinary arsenic concentration in late gestation, whereas the risk for diarrhea was more pronounced if exposure was based on urinary arsenic at early gestational (Table 3).

In the stratified analyses (by infant age, maternal education, socioeconomic status by asset index, and maternal BMI), we observed a significant risk increase for LRTI in all strata. However, the arsenic-related risk increase was more pronounced for infants of mothers with no formal education and low socioeconomic condition (Table 4). We observed no effect modification of arsenic exposure by these variables when tested in the model with interaction term (*p* > 0.10) (data not shown).

## Discussion

To our knowledge, this is the first prospective cohort study evaluating the association between prenatal arsenic exposure and the occurrence of LRTI and diarrhea, two of the most common causes of severe morbidity and mortality during infancy in low-income countries. These two conditions have been estimated to cause 50% of deaths among children < 5 years of age (Rudan et al. 2007). We observed a dose-dependent increased risk of LRTI and diarrhea in relation to prenatal arsenic exposure. Given that background and the magnitude of arsenic exposure in Bangladesh, our findings are of important public health significance.

In this community-based prospective study, the strengths were a relatively large sample size, objective measures of individual exposure based on urinary arsenic concentrations twice during pregnancy, prospectively collected outcome data, and availability of data on important covariates to adjust for possible confounding. Data were also collected over an entire calendar year, which would reduce any influence by seasons. We minimized measurement errors through the use of standardized protocols to maintain the quality of urine samples from collection to laboratory analyses. Potential limitations include outcome classification based on mothers’ reports of symptoms and signs, lack of measurements of infant exposure to arsenic, and a lack of information about other potentially toxic substances in water and food.

The measured urinary arsenic concentrations reflect exposure to inorganic arsenic from all sources of water, as well as from food during pregnancy. The analytical method used in the analysis, hydride generation atomic absorption spectroscopy, is specific for the sum of inorganic arsenic and its metabolites. Earlier studies have shown that very small amounts of arsenic pass to breast milk (Concha et al. 1998; Fangstrom et al. 2008). In the same study area as this study, arsenic levels in urine samples collected in infants at 3 months of age were very low (median, 1 μg/L) compared with maternal concentrations (Fangstrom et al. 2008), particularly among infants who were exclusively breast-fed. About 50% of the infants of the MINIMat cohort were exclusively breast-fed for 4–6 months (Eneroth et al. 2009) and partially breast-fed for > 2 years (Saha et al. 2008). Therefore, we assume that the observed associations with morbidity in infancy probably were consequences of *in utero* exposure to arsenic, possibly in combination with maternal toxicity during pregnancy, with indirect effects on the fetus.

Identifying cases based on reported symptoms through regular home visits is a common way of collecting information on acute respiratory tract infection (ARI) and diarrhea in epidemiologic studies and demographic and health surveys in developing countries, where most cases are not reported to health facilities (Feikin et al. 2010). ICDDR,B initiated an ARI surveillance in 1988, and case identification was carried out by active surveillance with biweekly home visits of CHRWs for initial 2 years (Fauveau et al. 1992) and thereafter by passive surveillance. Therefore, the knowledge of key symptoms of ARI may be relatively high among the study population. Further, including fast breathing and chest in-drawing as signs of LRTIs increases the positive predictive value of the screening questions (Lanata et al. 2004). Health surveillance in the study area has also focused on diarrheal diseases, so the mothers were likely to correctly report symptoms and signs of diarrhea as well. Even if there are errors in self-reported symptoms of these disease groups, there is no reason to believe that such misclassifications would be differential in relation to arsenic exposure levels.

Some previous studies have reported respiratory symptoms and signs such as chronic cough, bronchitis, and bronchiectasis, in relation to drinking water with high arsenic levels (Islam et al. 2007; Mazumder et al. 2005; Milton and Rahman 2002). In a review paper on the burden of pneumonia, the rate of pneumonia was reported 0.1–1.2 episodes/person-year among children < 5 years of age (Singh 2005). The rate in our study is higher than that, which may be attributable to the age range of infancy in our study and/or the widespread exposure to arsenic in the study population. Another report from rural Bangladesh showed an incidence rate of diarrhea of 4.25 episodes per child-year, which is similar to the rate we observed in our study (Pathela et al. 2006).

A possible causal pathway for an effect of arsenic exposure on incidence of LRTI and diarrhea is through immunosuppression, as suggested by several studies mentioned above (Banerjee et al. 2009; Conde et al. 2007; Lemarie et al. 2006; Patterson et al. 2004; Selgrade 2007). Indeed, in a subset of mothers in this cohort, we found that arsenic exposure in pregnancy, particularly in early pregnancy, was associated with fewer T cells (CD3^+^ cells) in the placenta (Ahmed et al. 2011). Arsenic induces reactive oxygen species, as also observed in mothers of the present cohort (Ahmed et al. 2011; Engström et al. 2010), and oxidative stress is known to affect the immune system (Knight 2000; NRC 2001). In another study in our cohort of women, we observed an association between prenatal arsenic exposure and decreased thymus size in the offspring (Moore et al. 2009), as well as secretion of trophic factors such as lactoferrin and interleukin-7 in breast milk (Raqib et al. 2009).

From a public health point of view, the observed increased risks of LRTI and diarrhea morbidity are noticeable, considering the effect sizes and extent of arsenic exposure worldwide. For example, an RR of 1.69 for LRTI with high arsenic exposure and an incidence rate of 2.96 LRTI episodes/person-year indicated about 2 additional episodes/person-year among the highly exposed relative to those with low exposure. An RR of 1.20 for diarrhea with high arsenic exposure and an incidence rate of 4.01 diarrhea episodes/person-year indicated < 1 additional episode/person-year among the highly exposed relative to those with low exposure.

We observed significant risk increase of LRTI and diarrhea morbidity irrespective of sociodemographic factors or nutritional status of the women. However, further evaluation is needed in a larger sample to evaluate the possible effect modification, because the RRs seemed to be more pronounced in low social strata. The possibility of residual confounding in our analyses cannot be completely ruled out, but we believe it is low given adjustment for potential confounding by relevant covariates. Measurement of arsenic exposure in early childhood is required in future studies to evaluate the association between arsenic exposure and morbidity in older children.

## Conclusions

We have shown that prenatal arsenic exposure was associated with increased risk of LRTI and diarrhea morbidity of the offspring during infancy. Taken together with earlier reports of effects on health, the findings strongly emphasize the need for a lowering of arsenic exposure via drinking water.

## Figures and Tables

**Table 1:** Concentration of urinary arsenic in early and late gestational periods (GW8 and GW30) among the 2002–2003 pregnancy cohort in Matlab, Bangladesh.

Statistic	Urinary arsenic concentration (μg/L)^a^		Average of GW8 and GW30^b^
	GW8	GW30	
Mean ± SD	152 ± 175	166 ± 196	159 ± 163
Median	79	80	94
25th percentile	36	41	43
75th percentile	210	225	220
Lowest	1	2	6
Highest	1,211	1,440	977

a Sum of inorganic and methylated arsenic species.

b Mean of the two urinary arsenic concentrations (in early and late gestation).

**Table 2:** Background characteristics and their associations with mothers’ urinary arsenic concentrations and reported symptoms of LRTI and diarrhea among the infants of the 2002–2003 pregnancy cohort in Matlab, Bangladesh.

		Arsenic level					
		Median (μg/L)^a^	*p*-Value	LRTI		Diarrhea	
Characteristic	*n* (%)			RR (95% CI)	*p*-Value	RR (95% CI)	*p*-Value
Age (years)							
< 20^b^	182 (11.7)	118	0.263	1		1	
20–24	442 (28.9)	96		0.81 (0.65–1.01)	0.059	1.15 (0.94 –1.41)	0.174
25–34	744 (47.9)	92		0.99 (0.81–1.21)	0.954	1.26 (1.04–1.52)	0.019
≥ 35	184 (11.9)	80		1.01 (0.78–1.30)	0.936	1.35 (1.07–1.70)	0.010

Parity							
0^b^	502 (32.4)	91	0.572	1		1	
1	399 (25.7)	93		1.13 (0.94–1.35)	0.186	1.15 (0.99–1.33)	0.066
2–3	330 (21.3)	100		1.36 (1.14–1.62)	0.001	1.17 (1.00–1.36)	0.051
≥ 4	320 (20.6)	101		1.40 (1.17–1.67)	< 0.001	1.29 (1.11–1.50)	0.001

Education (years)							
0^b^	390 (25.1)	107	0.001	1		1	
1–5	479 (30.9)	103		0.85 (0.72–1.00)	0.050	1.01 (0.88–1.17)	0.832
> 5	683 (44.0)	83		0.72 (0.62–0.85)	< 0.001	0.83 (0.72–0.95)	0.008

Asset index							
1 (poorest)^b^	344 (22.2)	124	< 0.001	1		1	
2	328 (21.1)	103		0.76 (0.64–0.91)	0.002	0.79 (0.67–0.93)	0.004
3	284 (18.3)	104		0.67 (0.55–0.81)	< 0.001	0.73 (0.62–0.87)	< 0.001
4	284 (18.3)	84		0.54 (0.44–0.66)	< 0.001	0.71 (0.60–0.84)	< 0.001
5 (richest)	312 (20.1)	65		0.46 (0.37–0.57)	< 0.001	0.69 (0.59–0.82)	< 0.001

Height (m)							
< 1.50^b^	787 (50.7)	95	0.847	1		1	
≥ 1.50	765 (49.3)	94		1.06 (0.94–1.21)	0.342	1.03 (0.93–1.15)	0.540

BMI (kg/m^2^)							
< 18.5^b^	464 (30.0)	110	0.026	1		1	
18.5–24	1,005 (64.5)	91		0.87 (0.76–1.00)	0.057	0.95 (0.85–1.08)	0.444
≥ 25	79 (5.1)	75		0.62 (0.43–0.88)	0.008	1.03 (0.79–1.33)	0.838

Birth weight of infant (g)							
< 2,000^b^	58 (3.7)	92	0.588	1		1	
2,000–2,499	421 (27.1)	88		0.86 (0.61–1.22)	0.405	0.99 (0.73–1.35)	0.955
≥ 2,500	1,072 (69.1)	98		0.85 (0.61–1.18)	0.328	0.95 (0.71–1.28)	0.727

Gestational age at birth (weeks)							
< 37^b^	181 (11.7)	103	0.295	1		1	
≥ 37	1,371 (88.3)	93		0.83 (0.67–0.98)	0.030	0.83 (0.71–0.98)	0.028

Sex of infant							
Male^b^	804 (51.8)	92		0.534	1	1	
Female	748 (48.2)	97		0.83 (0.73–0.94)	0.004	0.93 (0.83–1.04)	0.212

a Mean urinary arsenic concentrations in early and late gestation; sum of inorganic and methylated arsenic species.

b Reference group.

**Table 3:** RRs for episodes of LRTI and diarrhea among the infants in relation to maternal arsenic exposure in pregnancy: 2002–2003 pregnancy cohort in Matlab, Bangladesh.

Arsenic levels in quintiles (μg/L)^a^	Person-weeks of recall	LRTI			Severe LRTI			Diarrhea		
		No. of episodes	RR (95% CI)	Adjusted RR (95% CI)^b^	No. of episodes	RR (95% CI)	Adjusted RR (95% CI)^b^	No. of episodes	RR (95% CI)	Adjusted RR (95% CI)^b^
Average^c^										
< 39^d^	3,380	134	1		107	1		238	1	1
39–64	3,394	172	1.28 (1.02–1.60)	1.28 (1.02–1.61)	143	1.33 (1.04–1.71)	1.33 (1.03–1.71)	236	0.99 (0.082–1.18)	0.99 (0.83–1.19)
65–132	3,309	185	1.41 (1.13–1.76)	1.33 (1.07–1.67)	148	1.41 (1.10–1.81)	1.31 (1.02–1.69)	230	0.99 (0.82–1.18)	0.96 (0.80–1.15)
133–261	3,198	215	1.69 (1.37–2.10)	1.57 (1.27–1.96)	173	1.71 (1.34–2.17)	1.54 (1.21–1.97)	291	1.29 (1.09–1.53)	1.25 (1.05–1.48)
≥ 261	3,269	237	1.83 (1.48–2.26)	1.69 (1.36–2.09)	177	1.71 (1.34–2.17)	1.54 (1.21–1.97)	283	1.23 (1.03–1.46)	1.20 (1.01–1.43)

GW8										
< 32^d^	3,373	155	1	1	120	1	1	210	1	1
32–56	3,407	167	1.07 (0.86–1.33)	1.08 (0.86–1.34)	141	1.16 (0.91–1.49)	1.16 (0.91–1.49)	254	1.20 (1.00–1.44)	1.20 (1.00–1.44)
57–116	3,335	179	1.17 (0.94–1.45)	1.12 (0.90–1.39)	144	1.21 (0.95–1.55)	1.14 (0.89–1.45)	252	1.21 (1.01–1.46)	1.19 (0.99–1.43)
117–247	3,152	220	1.52 (1.24–1.86)	1.43 (1.17–1.77)	169	1.51 (1.19–1.90)	1.39 (1.10–1.76)	268	1.36 (1.14–1.63)	1.33 (1.11–1.60)
≥ 248	3,280	222	1.47 (1.20–1.81)	1.37 (1.11–1.68)	174	1.49 (1.18–1.88)	1.34 (1.06–1.70)	294	1.44 (1.20–1.72)	1.40 (1.17–1.68)

GW30										
< 36^d^	3,464	139	1		115	1		242	1	1
36–61	3,290	184	1.39 (1.12–1.74)	1.33 (1.06–1.66)	159	1.45 (1.14–1.85)	1.37 (1.08–1.74)	259	1.12 (0.94–1.34)	1.12 (0.94–1.33)
62–114	3,378	184	1.36 (1.09–1.69)	1.30 (1.04–1.62)	134	1.19 (0.93–1.53)	1.13 (0.88–1.45)	237	1.00 (0.84–1.20)	0.99 (0.83–1.19)
115–268	3,249	193	1.48 (1.19–1.84)	1.38 (1.11–1.72)	161	1.49 (1.17–1.90)	1.35 (1.06–1.72)	261	1.15 (0.96–1.37)	1.11 (0.93–1.32)
≥ 269	3,169	243	1.91 (1.55–2.35)	1.74 (1.41–2.14)	179	1.70 (1.34–2.15)	1.50 (1.18–1.90)	279	1.27 (1.06–1.50)	1.21 (1.02–1.44)

a Sum of inorganic and methylated arsenic species.

b Test for linear trend *p* < 0.05 for all exposures levels (GW8, GW30, and average) except diarrhea for GW30, adjusted for mother’s education, asset index, parity, BMI, gestational age, and infant’s sex.

c Mean urinary arsenic concentrations at GW8 and GW30.

d Reference group.

**Table 4:** RR (95% CI) of LRTI in relation to maternal arsenic exposure in pregnancy (mean of urinary arsenic measures at GW8 and GW30) stratified by infants’ age, mothers’ education level, socioeconomic status, and BMI in the in 2002–2003 pregnancy cohort in Matlab, Bangladesh.

	Infant’s age^b^		Education^b^		Socioeconomic status^b^,^c^		BMI (kg/m^2^)^b^	
Arsenic level^a^	0–5 months	6–11 months	No schooling	Attended school	Low	High	< 18.5	≥ 18.5
< 39^d^	1	1	1	1	1	1	1	1
39–64	1.11 (0.81–1.51)	1.47 (1.05–2.05)	2.68 (1.65–4.35)	0.97 (0.75–1.27)	1.83 (1.28–2.62)	0.85 (0.58–1.25)	1.38 (0.91–2.10)	1.22 (0.93–1.60)
65–132	1.23 (0.90–1.66)	1.48 (1.06–2.07)	2.74 (1.64–4.655)	1.08 (0.94–1.38)	1.79 (1.26–2.55)	1.08 (0.73–1.58)	1.19 (0.78–1.81)	1.41 (1.08–1.83)
133–261	1.31 (0.97– 1.78)	1.89 (1.38–2.60)	3.28 (2.04–5.27)	1.20 (0.93–1.55)	2.42 (1.73–3.37)	1.23 (0.85–1.80)	1.73 (1.17–2.56)	1.51 (1.16–1.96)
262–977	1.57 (1.18–2.10)	1.81 (1.32–2.49)	2.41 (1.48–3.92	1.53 (1.20–1.94)	2.25 (1.61–3.14)	1.57 (1.09–2.26)	1.92 (1.31–2.80)	1.54 (1.19–2.01)

a Sum of inorganic and methylated arsenic species.

b Adjusted for all the variables in the table except the one used for stratification.

c Socioeconomic status: low, asset index 1 or 2; high, asset index 4 or 5.

d Reference group.
